# The rise of generative AI and enculturating AI writing in postsecondary education

**DOI:** 10.3389/frai.2023.1259407

**Published:** 2023-08-10

**Authors:** Isabel Pedersen

**Affiliations:** Faculty of Social Science and Humanities, Ontario Tech University, Oshawa, ON, Canada

**Keywords:** generative AI, education, culture, AI writing, hype

## 1. Introduction

OpenAI'S release of ChatGPT shocked the public not only because so many people adopted it so quickly, but also because generative AI challenges the reverence society has for the *act* of writing. The rise of AI writing tools instigates a cultural moment that is difficult to measure. Universities are compelled to adapt to generative AI as a phenomenon before there is agreement upon how AI writing should be used or even valued by society, causing policymaking to be reactive. While higher education faculty members and professionals in teaching and learning are largely concentrating on whether the technology is factually correct or not in the writing it produces, or whether a student might be cheating, few concentrate on its threat to “writing culture” as an aspect of society at large. This opinion piece argues that the hype surrounding generative AI writing is a response to its cultural disruption. It suggests that higher education will need to decide if using AI writing will be *valued* as an aesthetic or professional practice and a means to garner what social theorist Pierre Bourdieu calls “cultural capital” (Bourdieu, 1986). In sum, will we start to recognize AI writing as *good writing*, or those use it as *good writers* demonstrating a shift in cultural attitudes and shared values?

## 2. Issues

There are four main issues at the moment relevant to the cultural adaptation to generative AI writing and the hype surrounding it. First, the speed of adoption has been dramatic. Generative AI has emerged more quickly than any current Internet platform or service. “ChatGPT gained one million users just 5 days after launching in November of last year [2022]. The conversational AI bot that can produce human-like text has been put to all kinds of uses, from writing short stories, prose, music and term papers to programming basic code, solving math problems and doing translations” (Buchholz, 2023). Education studies researchers have started to amass work geared to using generative AI in higher education pedagogy, developing digital and AI literacy, and making practical suggestions for institutions starting to use AI (Duin and Pedersen, 2021). However, research and commentary surrounding the cultural process of adapting to generative AI, and its effect on higher education, has not been addressed. How do we value and judge whether generative AI compositions are good? Following Bourdieu, “many literacy researchers accept that literacy is a form of cultural capital” (Walsh and Apperley, 2009, p. 5); therefore, if one no longer proves that one is a literate person through writing, how will culture accommodate this transformation? The rapid process of emergence by generative AI, which continues to evolve, has not undergone cultural assimilation.

Second, generative AI is shocking because its writing is stylistically correct, like that of a human. More to the point, one may never be able to detect if a machine or human wrote it. While there are precursors to autonomous writing, no technology has been introduced to society that is good enough to write *for* people. A Generative Pretrained Transformer (GPT) is a type of large language model (LLM) that uses machine learning to generate text that appears human-like. GPTs can generate new text based on the input (prompts) they receive. They are trained on a large corpus of text data and they are called transformers “because they use a transformer based neural network architecture to process input text and generate output text,” (Larsen and Narayan, 2023) that appears novel, just like humans create novel responses in their writing. Generative AI then threatens the appearance of human creativity. While it is known that “even if researchers trained these systems solely on peer-reviewed scientific literature, they might still produce statements that were scientifically ridiculous” (Metz, 2023), it does not change the unsettling fact that the form or style of writing no longer needs a human author.

Third, generative AI is designed to interact with humans through autonomous agents that address people as if they are other people. The conflict for educators lies in the suggestion that a personified generative AI appears to take on the work itself. Put another way, rather than using other forms of human-computer interaction, generative AI companies choose conversational AI agents to interact with people in written textual exchanges. The simplicity of using them is beyond basic, simplifying the complex layers of algorithms operating to produce the results. Dignum (2019) defines autonomy as “the capacity of an agent to act independently and to make its own free choices” (p. 17). She opines that autonomy “is both seen as a synonym for intelligence, as well as that characteristic of AI that people are most concerned about” (Dignum, 2019, p. 18). Artificial Intelligence companies will only continue to produce autonomous conversational AI agents that further mimic human intelligence (Duin and Pedersen, 2021, 2023). Higher education has a significant task ahead because ChatGPT is only the beginning.

Fourth and most significant, generative AI produces biased and discriminatory results due to language incorporated in the training sources (Duin and Pedersen, 2021, p. 18). Hu (2020) writes of GPT technologies “since it is a black box, we cannot easily predict or control the text it generates” and “an unsupervised GPT-3 could generate text that is biased or hurtful.” While generative AI companies discuss the problem of biased and discriminatory results, there is still no solution to eliminate its ability to produce it.

## 3. Discussion

Generative AI challenges *writing* as a highly valued artform, human skill, and professional practice. As a medium of communication, writing is used for art (e.g., novels, plays, poetry, screen plays); it is used to express one's creativity, a value judged by historical conventions and agreed upon societal expectations. We use *writing* to judge students, academics, and people; if one is considered a good writer, one gains legitimacy, garnering better opportunity to obtain resources. However, if everyone can be a good writer by using ChatGPT, society's values are forced to dramatically change, and that is the transformation occurring now. Generative AI writing is changing how people are judged, not only how writing is judged.

Writing is the primary means of human communication used for the purposes of information exchange (e.g., letters), proof (e.g., witness statements in legal contexts), and persuasion (e.g., sales, politics, leadership). However, in much more personal terms, we use writing for self-reflection (e.g., diaries, autobiography) and to remember our pasts. It can be used as a therapeutic apparatus and a way to express trauma. Cultural admiration for writing is one reason we use it for education or require it to serve as an outcome of education and a means to be rewarded for work completed (e.g., well-written documents across genres serve as proof of *being* educated). If an autonomous agent writes for a human in these capacities, the writing is judged as inauthentic. Its value is not yet agreed upon. Mostly, generative AI writing is debated in the terms of students' academic misconduct in a manner similar to plagiarism. Even if an instructor agrees to let students use it, there is often a process of disclosure implying that it might be shameful.

UNESCO's Futures of Education initiative makes the claim in 2021 that “the digital transformation of education continues to accelerate” (UNESCO, 2021). Also, recently, further disruption in the field of Education is taking place with the rise of AI teachers (Pedersen and Duin, 2022). However, the question over whether that digital acceleration will involve people *mostly* using automated writing tools to write is an unexpected turn in this current digital transformation. While the assessment of the value of ‘born digital' objects has been judged by fields such as digital humanities, digital cultural heritage studies, and digital media studies in the humanities for decades, it has been a shock to fields outside them, such as Education despite calls from UNESCO to prepare for it.

### 3.1. Conclusion

How should higher education respond to the use of generative AI for writing by students? Emergent generative AI tools need to be challenged by faculty members, instructors, administrators, and students for their ethical and cultural development in learning contexts, forcing them to go through an appropriate process of cultural adaptation. By doing so, further questions can come to the fore. Could generative AI writing tools be used to help those made vulnerable by digital divides acquire language skills as an apparatus for social change? Could it be used to level playing fields through language translation in classrooms? Could it make opaque concepts clear and approachable for learners excluded due to literacy issues? These are the kinds of important questions higher education needs to ask at this point of generative AI emergence.

## Author contributions

IP: Conceptualization, Writing–original draft.
